# Clearly different mechanisms of enhancement of short-lived Nef-mediated viral infectivity between SIV and HIV-1

**DOI:** 10.1186/s12985-014-0222-z

**Published:** 2014-12-18

**Authors:** Keisuke Harada, Nobutoki Takamune, Shozo Shoji, Shogo Misumi

**Affiliations:** Department of Pharmaceutical Biochemistry, Faculty of Life Sciences, Kumamoto University, 5-1Oe-Honmachi, Chuo-ku, Kumamoto, 8620973 Japan; Innovative Collaboration Organization, Kumamoto University, 2-39-1 Kurokami, Chuo-ku, Kumamoto, 8608555 Japan

**Keywords:** SIV, HIV, Nef, Retrovirus infectivity

## Abstract

**Background:**

One of the major functions of Nef is in the enhancement of the infectivity of the human and simian immunodeficiency viruses (HIV and SIV, respectively). However, the detailed mechanism of the enhancement of viral infectivity by Nef remains unclear. Additionally, studies of mechanisms by which Nef enhances the infectivity of SIV are not as intensive as those of HIV-1.

**Methods:**

We generated short-lived Nef constructed by fusing Nef to a proteasome-mediated protein degradation sequence to characterize the Nef role in viral infectivity.

**Results:**

The apparent expression level of the short-lived Nef was found to be extremely lower than that of the wild-type Nef. Moreover, the expression level of the short-lived Nef increased with the treatment with a proteasome inhibitor. The infectivity of HIV-1 with the short-lived Nef was significantly lower than that with the wild-type Nef. On the other hand, the short-lived Nef enhanced the infectivity of SIV_mac239_, an ability observed to be interestingly equivalent to that of the wild-type Nef. The short-lived Nef was not detected in SIV_mac239_, but the wild-type Nef was, suggesting that the incorporation of Nef into SIV_mac239_ is not important for the enhancement of SIV_mac239_ infectivity.

**Conclusions:**

Altogether, the findings suggest that the mechanisms of infectivity enhancement by Nef are different between HIV-1 and SIV_mac239_. Lastly, we propose the following hypothesis: even when the expression level of a protein is extremely low, the protein may still be sufficiently functional.

## Background

The negative regulatory factor (Nef), a 27–35-kDa non-enzymatic protein, is an accessory protein of human and simian immunodeficiency viruses (HIV and SIV, respectively) that enhances viral replication; it is associated with the pathogenesis induced by these viruses both in vivo and in vitro [1-4]. Furthermore, Nef has many functional motifs for contact to host proteins [5], through which it can serve as a molecular adaptor, and exert multiple functions, like the downregulation of CD4 and major histocompatibility complex (MHC) class I [6]. *N*-Myristoylation occurs at the *N*-terminus of Nef [7], whose posttranslational modification is essential for its multiple functions [6].

One of the major functions of Nef is in the enhancement of viral infectivity, which is independent of CD4 [8] or MHC class I [9] downregulation. Many studies have suggested that the Nef incorporation into virions or the association of virions with Nef is responsible for the enhancement of viral infectivity mediated by Nef [10-15]. Welker et al. estimated that approximately 10 Nef molecules are incorporated into HIV-1 virions [13], much fewer than the 275 molecules of another accessory protein, Vpr [16], which also has the capability to enhance viral infectivity [17,18]. In many reports [15,19-25], it has been suggested that Nef incorporated into virions could exert its action in the early steps of infection, although the molecular mechanism of this phenomenon remains substantially unclear [26].

Although Nef is clearly one of the factors for the pathogenesis induced by SIV in vivo [3], cell culture studies of the role of Nef in the enhancement of the infectivity of SIV [27] are not as intensive as those of HIV-1 described above. We, therefore, examined whether the Nef incorporation into virions or the association of virions with Nef is also important for the enhancement of the infectivity of SIV_mac239_ as well as of HIV-1.

In this study, we generated short-lived Nef constructed by fusing Nef to a proteasome-mediated protein degradation sequence, by which the balance between the synthesis and degradation of Nef can be changed. Consequently, the apparent expression level of Nef fused to the degradation sequence becomes much lower than that of the wild-type Nef. Accordingly, the frequency of the incorporation of the short-lived Nef into vitions or the association of virions with the short-lived Nef would be lower than that with the wild-type Nef. Interestingly, the infectivity of SIV_mac239_ with the short-lived Nef was equivalent to that with the wild-type Nef. On the other hand, the infectivity of HIV-1_NL4–3_ with the short-lived Nef was significantly lower than that with the wild-type Nef. These results suggest that the mechanisms of the Nef-mediated enhancement of infectivity are different between SIV and HIV-1.

## Results

### Construction of short-lived Nef expression vector

Some degradation signals conferring instability on proteins have been found, which include the CL peptide [28] and a murine ornithine decarboxylase (MODC) PEST region [29]. These signals induce a rapid protein degradation mediated by a proteasome, in which the CL peptide requires ubiquitination prior to degradation, whereas the PEST sequence requires no ubiqutination [14]. The CL peptide and PEST sequence convert stable proteins into unstable ones by attachment as fusion proteins [28,30-32], whose apparent expression levels could be much lower than those of the original proteins [30-33]. In this study, we utilized a combination of two protein degradation sequences of the CL peptide and PEST sequence, namely, the CP sequence, to generate short-lived Nef. We hypothesized that the apparent expression level of short-lived Nef is lower than that of wild-type Nef, which we evaluated to determine whether the Nef incorporation into virions is associated with the enhancement of the infectivity of SIV_mac239_.

**Figure 1:**
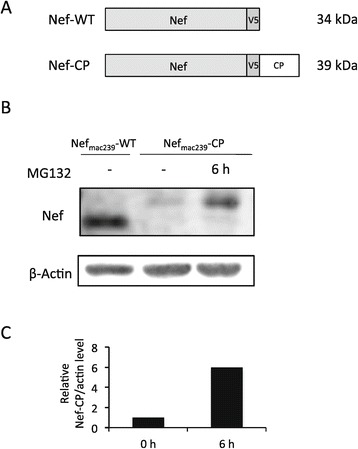
**Construction of short-lived Nef expression vector.** Schematic representations of Nef-WT and Nef-CP used in this study **(A)**. HEK293 cells were transfected with pNef_mac239_-WT or pNef_mac239_-CP. After a 48 h cultivation, HEK293 cells transfected with pNef_mac239_-CP were treated with 20 μM MG132 for 0 or 6 h. The cells were collected and subjected to SDS-PAGE and western immunoblot analysis to detect Nef_mac239_-WT and Nef_mac239_-CP using the anti-V5 antibody. β-Actin was also detected using the anti-β-actin antibody, as described in Materials and Methods **(B)**. The relative band intensities of Nef_mac239_-CP and β-actin were quantified using Fujifilm Image Gauge software. The expression levels of Nef_mac239_-CP relative to those of β-actin treated with and without MG132 were calculated using the obtained intensities and compared **(C)**.

To detect Nef by western immunoblot analysis under the same conditions, the V5 epitope was appended at the C-terminus of Nef to preserve the *N*-myristoylation site of Nef, which is an essential posttranslational modification for Nef functions [6,7]. The CP sequence was fused at the C-terminus of V5-tagged Nef (Figure 1A). Both wild-type (WT) Nef and CP-fused Nef were expressed under the control of the cytomegalovirus (CMV) promoter. To verify the effect of the fused CP sequence on Nef expression level, HEK293 cells were transfected with plasmids encoding Nef_mac239_-WT or Nef_mac239_-CP, which were from the SIV_mac239_ strain, and cultured for 72 h. The expression level of each Nef was evaluated by immunoblot analysis using an anti-V5 antibody. As shown in the two lanes on the left of Figure 1B, the apparent expression level of Nef_mac239_-CP was expectedly much lower than that of Nef_mac239_-WT. Uniform sample loading was confirmed by western immunoblot analysis of actin. As expected, the mobility of Nef_mac239_-CP was slightly but clearly lower than that of Nef_mac239_-WT with the fused CP sequence. To confirm that the relatively low expression of level of Nef_mac239_-CP is due to the introduction of the property of an extremely short life induced by proteasome-mediated degradation, treatment with the proteasome inhibitor MG132 for 6 h before the cell lysis was carried out, and Nef_mac239_-CP was then detected by western immunoblot analysis. As shown in the two lanes on the right of Figure 1B, the apparent expression level of Nef_mac239_-CP increased time-dependently about 6-fold in the cells treated with MG132 for 6 h relative to the level in the untreated cells (Figure 1C). This result validates that the relatively low expression level of Nef_mac239_-CP is due to its short-life property induced by fusing Nef to the CP sequence.

### Difference in incorporation level in virions between Nef_mac239_-WT and Nef_mac239_-CP

**Figure 2:**
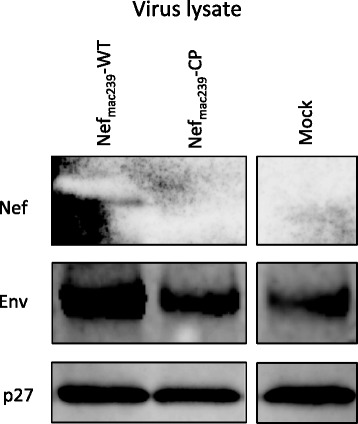
**Difference in incorporation level in virions between Nef**_**mac239**_ **-WT and Nef**_**mac239**_ **-CP.** pBRmac239Δ*nef*/*luc* was cotransfected into HEK293 cells with either pNef_mac239_-WT, pNef_mac239_-CP, or an empty vector. For virion preparation, the supernatants were subjected to ultracentrifugation. Each viral pellet was then subjected to SDS-PAGE and western immunoblot analysis to detect Nef, Env, and p27, as described in Materials and Methods. The mock sample was electrophoresed on a lane distant from two lanes of the Nef_mac239_-WT and Nef_mac239_-CP.

Next, we examined the incorporation level of Nef_mac239_-CP in the virions of SIV_mac239_. The *nef*-defective SIV_mac239_ provirus with the reporter gene was cotransfected into HEK293 cells with or without the plasmid encoding Nef_mac239_-WT or Nef_mac239_-CP. After a 72 h cultivation, the supernatants were collected by ultracentrifugation and used in the viral preparation, as described in Materials and Methods. The viruses were lysed and subjected to immunoblot analysis to detect Nef. Uniform virion loading was confirmed by western immunoblot analysis of the SIV p27 core antigen. As shown in the top panel of Figure 2, Nef_mac239_-WT was detected in SIV_mac239_. However, under the same conditions, Nef_mac239_-CP was not detected in SIV_mac239_, as in the mock transfection control. This result clearly indicates that the incorporation level of Nef_mac239_-WT is higher than that of Nef_mac239_-CP.

HIV-1 Nef enhances the incorporation of the envelope protein into virions [34]. Accordingly, we simultaneously examined the incorporation level of the envelope protein SIV_mac239_ virions. Western immunoblot analysis of the viral envelope protein was performed. As shown in the middle panel of Figure 2, the incorporation level of the envelope protein in SIV_mac239_ virions with Nef_mac239_-CP, which was equivalent to that of the mock transfection control, was clearly lower than that with Nef_mac239_-WT. Altogether, the phenotype of SIV_mac239_ virions with Nef_mac239_-CP was similar to that without Nef_mac239_.

### Comparison of effects of Nef-WTs and Nef-CPs on enhancement of SIV_mac239_ infectivity

**Figure 3:**
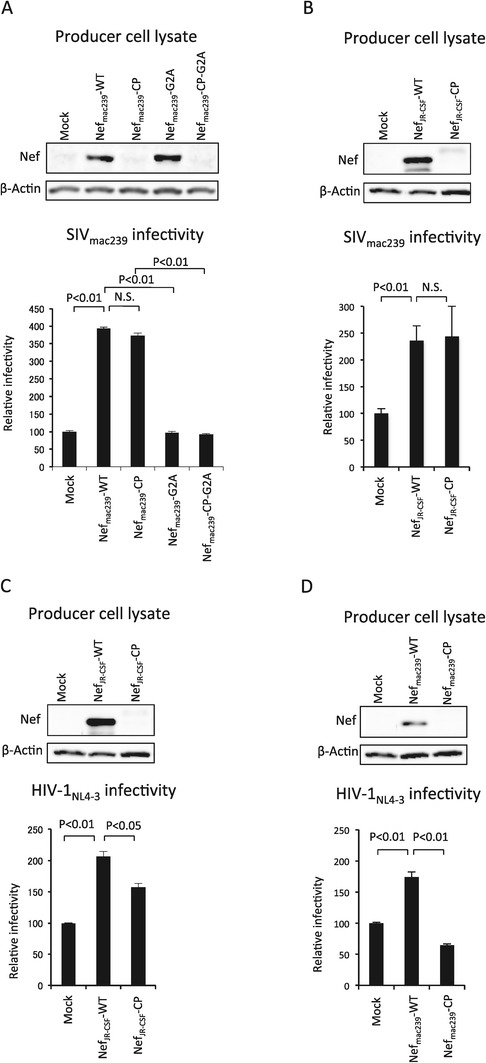
**Comparison of effects of Nef-WTs and Nef-CPs on enhancement of viral infectivity.** pBRmac239Δ*nef*/*luc* was cotransfected into HEK293 cells with either pNef_mac239_-WT, pNef_mac239_-CP, pNef_mac239_-G2A, pNef_mac239_-CP-G2A, or an empty vector **(A)**. pBRmac239Δ*nef*/*luc* was cotransfected into HEK293 cells with either pNef_JR-CSF_-WT, pNef_JR-CSF_-CP, or an empty vector **(B)**. pNL-CHΔ*env*Δ*nef*/*luc* and psvJR-FLenv were cotransfected into HEK293 cells with either pNef_JR-CSF_-WT, pNef_JR-CSF_-CP, or an empty vector **(C)**. pNL-CHΔ*env*Δ*nef*/*luc* and psvJR-FLenv were cotransfected into HEK293 cells with either pNef_mac239_-WT, pNef_mac239_-CP, or an empty vector **(D)**. After a 72 h cultivation, the cells were collected and subjected to SDS-PAGE and western immunoblot analysis to detect Nef and β-actin, as described in Materials and Methods (top panels). The supernatants were subjected to SIV p27 or HIV-1 p24 enzyme-linked immunosorbent assay (ELISA) and infectivity assay using MAGIC-5 cells, as described in Materials and Methods (bottom panels). Each bar represents the mean standard deviation (n = 3).

The viral infectivity enhanced by Nef_mac239_-CP was compared with that enhanced by Nef _mac239_-WT in SIV_mac239_. To examine the effects of Nef_mac239_-WT and Nef_mac239_-CP on SIV infectivity, HEK293 cells were cotransfected with a *nef*-defective SIV_mac239_ provirus with the luciferase reporter gene and the plasmid expressing Nef_mac239_-WT or Nef_mac239_-CP. Additionally, plasmids expressing nonmyristoylated G2A mutants, i.e., Nef_mac239_-G2A and Nef_mac239_-CP-G2A, were also used as nonfunctional Nef. The expression level of each Nef in the virus-producing cells was examined by western immunoblot analysis of the obtained cell lysate. Indicator cells, i.e., MAGIC-5 cells, were inoculated with each supernatant containing SIV_mac239_, and the luciferase activity of the MAGIC-5 cells was measured after a 48 h cultivation, as described in Materials and Methods. The MAGIC-5 cell line is a HeLa cell derivative modified to express CD4 (the primary receptor) and CCR5 (the coreceptor), respectively, for the infection by SIV_mac239_ and CCR5-tropic HIV-1. The infectivity of each virus was compensated for by the amount of each SIV p27 antigen. The expression level of each Nef_mac239_ derivative in each type of producer cell is shown in the top panel of Figure 3A. Uniform sample loading was confirmed by western immunoblot analysis of actin. The expression levels of Nef_mac239_-CP and Nef_mac239_-CP-G2A were much lower than those of Nef_mac239_-WT and Nef_mac239_-G2A.

The comparison of the relative infectivities of SIV_mac239_ produced from HEK293 cells expressing all the Nef_mac239_ derivatives is shown in the bottom panel of Figure 3A. The enhancement of the viral infectivity by Nef_mac239_-WT was observed as expected whereas the enhancement of the viral infectivity by Nef_mac239_-G2A, a nonmyristoylated and nonfunctional mutant, was not observed. Under this condition, SIV_mac239_ with Nef_mac239_-WT was about 4-fold more infectious than the virus without Nef. Unexpectedly, the enhancement of the viral infectivity by Nef_mac239_-CP was observed, although the apparent expression level of Nef_mac239_-CP was much lower than that of Nef_mac239_-WT. Interestingly, no significant difference in the enhancement of viral infectivity between Nef_mac239_-WT and Nef_mac239_-CP was observed. The enhancement by Nef_mac239_-CP was abolished by the nonmyristoylated G2A mutation, as in the case of the G2A mutation of Nef_mac239_-WT.

It was examined whether Nef from the HIV-1 strain can enhance SIV_mac239_ infectivity similarly to Nef_mac239_-CP. Nef from the JR-CSF strain of HIV-1, namely, Nef_JR-CSF_, was chosen in this study because the apparent expression level of Nef_JR-CSF_-WT is reported to be relatively higher than those of other HIV-1 strains. The top panel of Figure 3B shows the expressions of Nef_JR-CSF_-WT and Nef_JR-CSF_-CP in the lysate of the virus-producing cells determined by western immunoblot analysis. The apparent expression level of Nef_JR-CSF_-CP generated by fusion of the Nef to the CP sequence was much lower than that of Nef_JR-CSF_-WT, and the mobility of the weakly expressed Nef_JR-CSF_-CP was lower than that of Nef_JR-CSF_-WT as in the case of Nef_mac239_-CP (Figure 1B). Under this condition of the virus-producing cells, the infectivity of each produced SIV_mac239_ with or without Nef_JR-CSF_-WT or Nef_JR-CSF_-CP was examined. As shown in the bottom panel of Figure 3B, significant enhancement of the viral infectivity by Nef_JR-CSF_-WT and Nef_JR-CSF_-CP in comparison with that in the case of a virus without Nef was observed. Moreover, no significant difference in the enhancement of viral infectivity between Nef_JR-CSF_-WT and Nef_JR-CSF_-CP was observed, as in the case of Nef_mac239_-WT and Nef_mac239_-CP. Altogether, these results indicate that Nef-WTs and Nef-CPs show equivalent activities for the enhancement of SIV_mac239_ infectivity in the case of Nef from not only SIV_mac239_ but also HIV-1, even though the apparent expression levels were markedly different between Nef-WTs and Nef-CPs.

### Comparison of effects of Nef-WTs and Nef-CPs on enhancement of HIV-1 infectivity

Many studies have suggested that the Nef incorporation into virions or the association of virions with Nef is responsible for the enhancement of the infectivity of HIV-1 [10-13,15]. Accordingly, it has been expected that the activity of Nef-CPs would be lower than that of Nef-WTs in HIV-1. The effects of Nef-WTs and Nef-CPs on HIV-1 infectivity were evaluated as follows. HEK293 cells were cotransfected with an *env-* and *nef*-defective HIV-1_NL4–3_ provirus with the luciferase reporter gene inserted into *nef*, a plasmid encoding a CCR5-tropic JR-FL envelope protein, and a plasmid encoding either Nef_JR-CSF_-WT, Nef_JR-CSF_-CP, Nef_mac239_-WT, or Nef_mac239_-CP. To examine the infectivity, MAGIC-5 cells were infected with the supernatants including CCR5-tropic HIV-1_NL4–3_ and incubated for 48 h. Then, luciferase activity as the indicator of infectivity was measured. The infectivity of each virus was compensated for by the amount of the p24 antigen of each virus. The top panel of Figure 3C shows the expressions of Nef_JR-CSF_-WT and Nef_JR-CSF_-CP in the virus-producing HEK293 cells determined by western immunoblot analysis. Uniform sample loading was confirmed by western immunoblot analysis of actin. As expected, the expression level of Nef_JR-CSF_-CP was much lower than that of Nef_JR-CSF_-WT, whose expression profile was the same as that of Nef_JR-CSF_-WT or Nef_JR-CSF_-CP expressed in SIV_mac239_-producing cells (Figure 3B). Under this condition of the virus-producing cells, the infectivity of HIV-1_NL4–3_ with Nef_JR-CSF_-WT was significantly much higher than that of HIV-1_NL4–3_ without Nef. On the other hand, the infectivity of HIV-1_NL4–3_ with Nef_JR-CSF_-CP was significantly lower than that of HIV-1_NL4–3_ with Nef_JR-CSF_-WT, which correlated with the expression levels of Nef_JR-CSF_-WT and Nef_JR-CSF_-CP.

Next, it was examined whether Nef_mac239_-CP enhances HIV-1_NL4–3_ infectivity. The top panel of Figure 3D shows the expression profiles of Nef_mac239_-WT and Nef_mac239_-CP in the lysate of the virus-producing cells by western immunoblot analysis. Uniform sample loading was confirmed by western immunoblot analysis of actin. The expression level of Nef_mac239_-CP was found to be much lower than that of Nef_mac239_-WT, showing the same expression profile as that of each Nef_mac239_ derivative expressed in the SIV_mac239_-producing cells (Figure 3A). Under this condition of the virus-producing cells, the infectivity of HIV-1_NL4–3_ with Nef_mac239_-WT was significantly much higher than that of HIV-1_NL4–3_ without Nef. On the other hand, the infectivity of HIV-1_NL4–3_ with Nef_mac239_-CP was significantly lower than that of HIV-1_NL4–3_ with Nef_mac239_-WT, which correlated with the expression levels of Nef_mac239_-WT and Nef_mac239_-CP.

Taken together, the above results indicate that the activity of enhancement of HIV-1 infectivity by Nef-CPs is significantly lower than that by Nef-WTs, which were from both SIV_mac239_ and HIV-1_JR-CSF_. The difference in this activity between Nef-CPs and Nef-WTs was considered to be associated with the difference in apparent expression level between Nef-CPs and Nef-WTs. These results for HIV-1 could support previous reports suggesting that the Nef incorporation into virions or the association of virions with Nef is responsible for the enhancement of the infectivity of HIV-1 [10-13,15]. Therefore, the substantial enhancement of infectivity of SIV_mac239_ by Nef-CP is again considered to be a unique property.

### Low expression level of Nef_mac239_ is not sufficient for the enhancement of SIV_mac239_ infectivity

It was indicated that Nef-CPs could enhance the infectivity of SIV_mac239_, whose apparent activity was equivalent to those of Nef-WTs, although the apparent expression levels of Nef-CPs were much lower than those of Nef-WTs (Figures 3A and B). Typically, the activities of Nef-CPs are lower than those of Nef-WTs for the enhancement of SIV_mac239_ infectivity, similarly to HIV-1 (Figures 3C and D). However, the profiles of the activities of Nef-WTs and Nef-CPs for SIV_mac239_ were unexpectedly but interestingly different from those for HIV-1. Here, we hypothesized that even a very low expression level of Nef is sufficient to enhance the infectivity of SIV_mac239_. To test this hypothesis, a plasmid DNA encoding for Nef_mac239_-WT was diluted to 1, 0.9, 0.5, 0.1, 0.05, and 0 μg, to which mock empty plasmid DNA was added to a total 1 μg. HEK293 cells were cotransfected with 1 μg of a plasmid DNA mixture containing the indicated Nef_mac239_-WT-encoding DNA and 1 μg of the *nef*-defective SIV_mac239_ provirus with the reporter gene. Moreover, HEK239 cells were cotransfected with 1 μg of plasmid DNA encoding for Nef_mac239_-CP and 1 μg of the *nef*-defective SIV_mac239_ provirus with the reporter gene for comparison of the effect on infectivity between Nef_mac239_-WT from DNA of various amounts and Nef _mac239_-CP from 1 μg of DNA.

**Figure 4:**
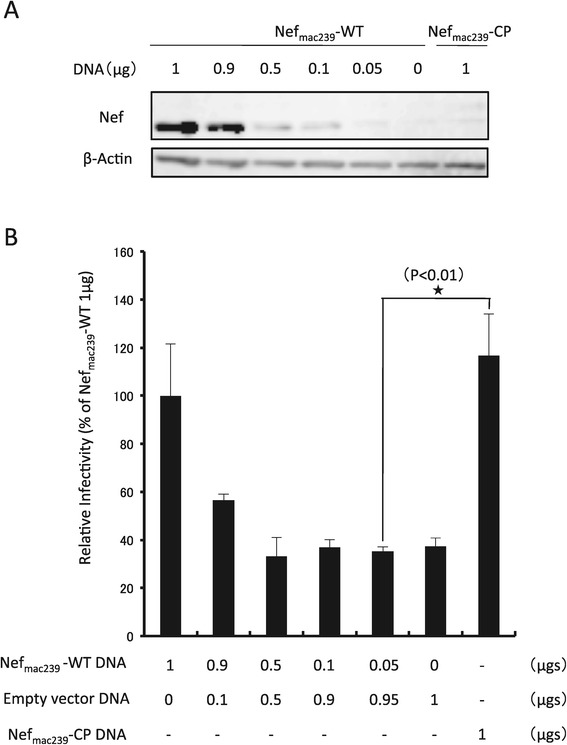
**Effect of low expression level of Nef**_**mac239**_ **on SIV**_**mac239**_ **infectivity.** pBRmac239Δ*nef*/*luc* was cotransfected into HEK293 cells with 1 μg of a mixture of pNef_mac239_-WT and an empty vector, at amounts of pNef_mac239_-WT in grams indicated. After a 72 h cultivation, the cells were collected and subjected to SDS-PAGE and western immunoblot analysis to detect Nef and β-actin, as described in Materials and Methods **(A)**. The supernatants were subjected to SIV p27 ELISA and infectivity assay using MAGIC-5 cells as described in Materials and Methods **(B)**. Each bar represents the mean standard deviation (n = 3).

At 72 h posttransfection and cultivation, the virus-producing cells were lysed and subjected to western immunoblot analysis to detect Nef_mac239_-WT and Nef_mac239_-CP. At the same time, the supernatants were evaluated for viral infectivity, as described above. As shown in Figure 4A, a graded decrease in the expression level of Nef_mac239_-WT was observed in a Nef_mac239_-WT encoding DNA dose-dependent manner. A very low expression level of Nef_mac239_-CP from 1 μg of DNA was observed, which was much lower than that of Nef_mac239_-WT from 1 μg of DNA and was almost equivalent to that of Nef_mac239_-WT from 0.05 μg of DNA. Under this condition of each virus-producing cell type, the infectivity of each SIV_mac239_ produced is shown in Figure 4B. A graded decrease in infectivity of the virus was observed in a transfected Nef_mac239_-WT encoding DNA dose-dependent manner. The infectivity of SIV_mac239_ produced from the cells transfected with 0.05 μg of DNA encoding Nef_mac239_-WT was significantly lower than that transfected with 1 μg of DNA encoding Nef_mac239_-CP. On the other hand, the infectivity of SIV_mac239_ produced by cells transfected with 1 μg of DNA encoding Nef_mac239_-WT was almost similar to that transfected with 1 μg of DNA encoding Nef_mac239_-CP, whose result was the same as that shown in Figure 3A. Taken together, these results indicate that such a low expression level of Nef_mac239_-WT, which is similar to that of Nef_mac239_-CP, is not sufficient to enhance the infectivity of SIV_mac239_.

## Discussion

Although the enhancement of viral infectivity by Nef is clear, the molecular mechanism by which Nef enhances the infectivity of HIV-1 and SIV remains unclear. In this study, it was clearly indicated that there is a significant difference between SIV_mac239_ and HIV-1_NL4–3_ in their response to the infectivity enhancement by Nef-CP, suggesting that the molecular mechanisms of Nef action differ between SIV_mac239_ and HIV-1_NL4–3_.

It has been reported that HIV-1 Nef enhances the incorporation of envelope proteins into virions, which is associated with the increase in HIV-1 infectivity [34]. Additionally, Nef incorporated into virions is suggested to directly enhance virion infectivity in the early steps of infection by HIV-1 [24,25]. In this study, it was observed that the amount of envelope protein of SIV_mac239_ without Nef_mac239_-WT was clearly lower than that with Nef_mac239_-WT, suggesting that Nef_mac239_ can also enhance the incorporation of envelope proteins into SIV_mac239_ as in the case of HIV-1 [34]. Thus, the amount of envelope protein of SIV_mac239_ with Nef_mac239_-CP was clearly lower than that with Nef_mac239_-WT, which was similar to that without Nef_mac239_-WT (Figure 2). On the other hand, the infectivity of SIV_mac239_ with Nef_mac239_-CP was interestingly equivalent to that with Nef_mac239_-WT (Figure 3A). Accordingly, it is suggested that the enhancement of the incorporation of the envelope protein into SIV_mac239_ by Nef_mac239_ is not associated with the enhancement of viral infectivity by Nef_mac239_. Additionally, the direct action of Nef incorporated to virions in the early step of SIV_mac239_ infection is suggested to be not essential for the enhancement of viral infectivity. Taken together, the results suggest that the molecular mechanisms of the Nef-mediated enhancement of viral infectivity are different between HIV-1 and SIV_mac239_. Furthermore, a mechanism by which Nef_mac239_ can enhance SIV_mac239_ infectivity inside virus-producing cells, which is not associated with the incorporation of envelope proteins into the virions, may exist.

The *N*-myristoylation of Nef is essential for the major functions of Nef [6]. The lack of effect of nonmyristoylated Nef_mac239-_CP-G2A on the enhancement of viral infectivity (Figure 3A) indicates that the function of Nef_mac239-_CP is also dependent on *N*-myristoylation and may be dependent on membrane localization. Since it was also confirmed that such a low expression level of Nef_mac239_-WT from 0.05 μg of DNA, similar to that of Nef_mac239_-CP from 1 μg of DNA, was not sufficient to enhance the infectivity of SIV_mac239_ (Figure 4), it is hypothesized that a protein synthesis process for producing a quorum of Nef_mac239_ molecules within a short time in SIV_mac239_-producing cells is required for the enhancement of viral infectivity even if the duration of the presence of each molecule of Nef_mac239_ is extremely short in the cell.

Lastly, we propose the following hypothesis: even when the expression level of a protein such as Nef-CP is extremely low, the protein may still be sufficiently functional; this novel and unique hypothesis might be greatly applicable in various fields such as biochemistry and cell biology. Thus, a rapid turnover by not only rapid protein degradation but also efficient production of a protein through a strong promoter activity in transcription or efficient translation might be essential for the protein to function sufficiently.

## Conclusion

The findings suggest that the mechanisms of the enhancement of viral infectivity by Nef are different between HIV-1 and SIV_mac239_. Additionally, we propose the following hypothesis: even when the expression level of a protein is extremely low, the protein may still be sufficiently functional.

## Methods

### Materials

The infectious HIV-1_NL4–3_ expression vectors pNL-CH [35] and psvJR-FLenv [36] were kindly gifted by Dr. Ron Swanstrom of the UNC Center for AIDS Research, University of North Carolina at Chapel Hill, Chapel Hill, NC. The infectious HIV-1 expression vector pYK-JRCSF was obtained from the NIH AIDS reagent program.

### Constructions of *nef-* and *env*-deficient and reporter-gene-introduced SIVmac239 and HIV-1 expression vectors

Firefly-luciferase-encoding DNA was amplified by PCR using a pGL4.14 [luc2/Hygro] vector (Promega, Madison, Wi) and inserted into the *nef*-coding region of pBRmac239, coding for SIV_mac239_ proviral DNA, as previously reported [37]. The *nef*-deficient and luciferase-gene-inserted SIV_mac239_ proviral DNA vector was named pBRmac239Δ*nef*/*luc*. To delete *env* of pNL-CH, the *Stu*I and *BsaB*I sites in *env* were digested by the corresponding enzymes and the linear DNA obtained was subjected to a ligation reaction, whose vector encoding the *env*-deficient proviral DNA was named pNL-CHΔ*env*. Firefly-luciferase-encoding DNA was amplified in PCR using a pGL4.14 [luc2/Hygro] vector and inserted into the *nef*-coding region using the *Xho* I site of pNL-CHΔ*env*, whose vector with the luciferase reporter gene and without *env* and *nef* was named pNL-CHΔ*env*Δ*nef*/*luc*.

### Nef expression vectors

Nef-coding DNAs were amplified by PCR using the corresponding proviral DNA templates (pBRmac239 for Nef_mac239_ and pYK-JRCSF for Nef_JR-CSF_) and subcloned into pcDNA3.1D/V5-His TOPO according to the manufacturer’s instructions (Invitrogen, Carlsbad, CA). A Gly^2^-to-Ala^2^ (G2A) mutation in *nef* was introduced using site-directed mutagenesis. To introduce the short-life property into the expressed Nef proteins, DNA encoding a sequence of the CL peptide and the PEST, namely, the CP sequence, was amplified from pGL4.78 [hRlucCP] Hygro (Promega, Madison, Wi). The DNA was then linked after the region of the V5 epitope tag encoding DNA using the *Age*I site of each Nef expression vector. All the constructed Nef expression vectors were named as follows: pNef_mac239_-WT, pNef_mac239_-CP, pNef_mac239_-G2A, pNef_mac239_-CP-G2A, pNef_JR-CSF_-WT, and pNef_JR-CSF_-CP.

### Viral preparation

For the preparation of SIV_mac239_, pBRmac239Δ*nef*/*luc* was transiently cotransfected with either pNef_mac239_-WT, pNef_mac239_-CP, pNef_mac239_-G2A, pNef_mac239_-CP-G2A, pNef_JR-CSF_-WT, pNef_JR-CSF_-CP, or an empty vector into HEK293 cells using Lipofectamine LTX reagent (Invitrogen, Carlsbad, CA). For the preparation of CCR5 tropic HIV-1_NL4–3_, pNL-CHΔ*env*Δ*nef*/*luc* and psvJR-FLenv were transiently cotransfected with either pNef_mac239_-WT, pNef_mac239_-CP, pNef_JR-CSF_-WT, pNef_JR-CSF_-CP, or an empty vector into HEK293 cells. At 72 h posttransfection, the supernatants were collected and filtered through a 0.45 μm filter and subjected to ELISA for p27 or p24 antigen or viral infectivity assay. For virion preparation, the supernatants were subjected to ultracentrifugation as previously described [38].

### Quantification of SIV p27 and HIV-1 p24 antigen in supernatant

Each cell-free supernatant was filtered using a 0.45-μm-pore-size filter and subjected to ELISA for SIV p27 or HIV p24 (ZeptoMetrix Corporation, Buffalo, NY), according to the manufacturer’s instructions.

### Measurement of viral infectivity

MAGIC-5 cells were inoculated with the supernatant as previously described [39]. After a 72 h cultivation, the cells were washed with PBS(−) and lysed with Cell Culture Lysis Reagent (Promega, Madison, WI). The luminescence of the firefly luciferase reporter was measured with a luciferase assay system (Promega, Madison, Wi) using a Wallac ARVO™ SX 1420 luminometer (Perkin-Elmer, Waltman, MA).

### Cell lysis and western immunoblot analysis

The cells were washed, lysed, and subjected to 5–20% polyacrylamide gradient sodium dodecyl sulfate-polyacrylamide gel electrophoresis (SDS-PAGE) and western blot analysis. The antibodies used in different immunoblottings were as follows: anti-V5 antibody (Invitrogen, Carlsbad, CA), anti-actin antibody (Oncogene, San Diego, CA), anti-gp130 SIV monoclonal antibody (Immuno Diagnostics, Inc., Woburn, MA), and anti-SIV_mac239_ monkey serum. Immunocomplexes were detected using appropriate peroxidase-conjugated secondary antibodies, followed by visualization by chemiluminescence detection (NEN Life Science Products, Boston, MA, USA) with LAS4000 (GE Healthcare, Buckingham, England). The intensities of the bands were quantified with Fujifilm Image Gauge Software.
